# Advanced practice nurses’ daily practices delivering primary care to residents in long-term care facilities: a qualitative study

**DOI:** 10.1186/s12875-024-02455-9

**Published:** 2024-06-08

**Authors:** Zúñiga Franziska, Teuscher Ramona, Stoll Hansruedi, Sailer Schramm Monique, Vökt Franziska, Kotkowski Kornelia

**Affiliations:** 1 Department of Public Health, Institute of Nursing Science, University of Basel, Bernoullistrasse 28, Basel, 4056 Switzerland; 2 Hirslanden, Salem Hospital, Bern, Switzerland; 3 Spitex Fricktal AG, Münchwilen, Switzerland; 4 MediZentrum Täuffelen AG, Täuffelen, Switzerland; 5 MediZentrum Schüpfen AG, Schüpfen, Switzerland; 6 Kantonalspital Aarau, Aarau, Switzerland

**Keywords:** Advanced practice nursing, Primary care, Qualitative research

## Abstract

**Background:**

Globally, there is a growing shortage of primary care professionals, including those who serve residents in long-term care facilities (LTCFs). In recent decades, numerous new care models have been implemented to improve these residents’ care. Many incorporate Advanced Practice Nurses (APNs) into interprofessional healthcare teams. In Switzerland, little is known about how these models function, and few facilities have integrated APNs. This study aims to explore the everyday practice of APNs employed at a medical centre in the Bernese Seeland region delivering care to LTC residents and collaborating with LTCFs staff.

**Methods:**

This qualitative study uses the “Interpretive Description” methodology, which builds on existing knowledge and examines phenomena interpreted through a social constructivist approach. We conducted six semi-structured individual interviews, one semi-structured focus group interview, and an examination of secondary data. Our thematic analysis followed Braun and Clarke’s guidelines for data analysis.

**Results:**

In LTCFs, APNs perform tasks similar to those of primary care physicians, e.g., patient visits and therapy adjustments, within the limits set by their supervising physicians. In addition, they contribute significantly to facility-wide quality improvement. We identified three fundamental elements for successful collaboration between APNs and LTCF staff: 1) clarifying roles and responsibilities; 2) establishing well-defined communication methods and pathways; and 3) building and maintaining trust. Together with LTCF staff, APNs provide multidimensional, person-centred care that focuses on medical, social, and nursing issues with the goal of maintaining the residents’ best possible quality of life.

**Conclusions:**

Our results suggest that integrating APNs into the LTCF care system improves care quality for residents and increases staff members’ job satisfaction.

**Supplementary Information:**

The online version contains supplementary material available at 10.1186/s12875-024-02455-9.

## Background

In recent decades, life expectancy has significantly increased worldwide. By 2045, it is estimated that one-third of the global population will be over 60 [1]. Ageing processes lead to decreases in people’s physiological reserves, coupled with increases in their functional limitations [1]. Neurocognitive disorders also increase with age. Due to the combination of physiological and cognitive limitations, older adults often require 24-h care in assisted living facilities (LTCFs) [2–4]. In the United States, between 2011 and 2014, over 1.4 million people lived in LTCFs [5]. In Switzerland, at the end of 2021, LTCFs housed a total of 86,969 residents [6].

In Switzerland, the medical care and treatment of these residents are mainly managed through primary medical care. Health care professionals in primary care aim to address population’s medical needs via treatment, promotion of good health, prevention of disease, rehabilitation, and palliation [7]. As primary care professionals play essential roles in ensuring that patients receive the appropriate treatment, they are vital to the healthcare system [7]. However, there is a growing worldwide shortage of healthcare professionals in primary medical care. This means that open positions for those who would normally work with LTCF residents are becoming more difficult to fill. Delays can lead to gaps in care [8]. In recent years, various new care models to improve the care of LTCF residents have been implemented in Europe and North America. Many of these models incorporate Advanced Practice Nurses (APNs) into interprofessional healthcare teams [9, 10].

APNs are registered nurses with master’s degrees in nursing science who possess advanced nursing and clinical competencies [11]. Their core competencies include direct clinical practice, which encompasses guidance, coaching, counselling, evidence-based nursing, clinical leadership, interprofessional collaboration, and support in ethical decision-making [12]. APNs provide continuous support to patients and their families in all areas of medical care, emphasizing the development of self-management strategies. Their clinical and nursing skills prepare them well to care for LTCF residents, many of whom live with chronic illnesses [9, 13].

In clinical practice, APNs assume a range of responsibilities, e.g., as Nurse Practitioners (NPs) or Clinical Nurse Specialists (CNSs) [14], depending on their competencies, activities and employment contexts. Internationally, NPs tend to work in hospitals, family practices, outpatient clinics and LTCFs. They can work alone or with other health professionals as members of interprofessional treatment teams, and often have their own patient bases. NPs are competent to perform clinical assessments, as well as prescribing laboratory tests or other diagnostic tests and even prescribing medications and treatments, depending on the regulatory context [14]. CNSs most often work in hospitals, health centres, and schools. Relying on an up-to-date understanding of the latest empirical evidence, they care for vulnerable patient populations and support interprofessional care teams [14]. Their duties vary internationally and regionally, depending on local legislation [15]. To date, APN roles have been developed and implemented in 27 countries, including parts of Europe, the USA, Canada, the UK, Australia, and New Zealand [16]. In the USA, Canada, Australia, and the UK, APNs often function as autonomous healthcare providers. In many countries, the implementation of their roles has partially filled gaps in primary medical care, especially in LTCFs [16]. In collaboration with primary care physicians, APNs increase LTCF residents’ quality of care and improve their medical outcomes [17–23]. Benefits have been observed regarding depression, urinary incontinence, pressure ulcers, and physical restraint use [4, 13, 17, 19, 21, 23]. Their presence also correlates with reduced hospital admissions and increased overall satisfaction of residents [4, 18, 23–27].

While many countries have already established regulatory frameworks for advanced clinical competencies, these are still under development in Switzerland [25, 28]. Here, recognition of the APN qualification has only been possible since 2020 and is provided by the private association APN-CH (where CH is the international two-letter ISO code of Switzerland) [28]. Incomplete political and financial prerequisites for the deployment of APNs hinder the development of their roles, especially in primary medical care, including in LTCFs [27]. Currently, only one Swiss canton has enacted regulations regarding NPs’ competencies, and reimbursement in primary care is limited to a fee-for-service system for physicians [28]. As a result, few primary care practices in Switzerland currently employ APNs [24, 28], and little is known about how such care models’ function. Initial studies suggest that APNs play crucial roles in LTCFs [28, 29], as is the case internationally [9, 13]. However, limited information is available regarding the tasks APNs perform in LTCFs and how they collaborate with LTCF staff. Accordingly, this study aimed to answer the following two questions: What care tasks do the APNs in a primary care medical centre perform for LTCF residents?; and How does the collaboration between APNs and LTCF staff in the care of residents work from the perspective of the entire treatment team (which includes LTCF staff like registered nurses and licensed practical nurses, LTCF directors, directors of nursing, geriatricians, and APNs)?

## Methods

### Study design

The current study follows Interpretive Description methodology, which guides the investigation of themes and patterns of subjective experiences of both the primary medical care centre and LTCF staff to generate new knowledge within a clinical context. Building on existing knowledge, this methodology examines and interprets phenomena using a social constructivist approach [30]. To explore the participants’ subjective experiences of practices, six semi-structured individual interviews, one semi-structured focus group interview were conducted, along with an examination of secondary data. A reflective thematic analysis according to Braun and Clarke was performed [31]. To present tasks and collaboration in a practical context, this approach involves identifying themes and illuminating them in new and meaningful ways [32].

### Research setting

The study takes place in a MediZentrum in the canton of Bern in Switzerland, which is part of a group of five MediZentrum centres in the Bernese Seeland and two in the Bernese Oberland regions. These MediZentrum are examples of new care models in primary care that employ APNs. Employees at these centres provide care to acutely and chronically ill individuals of all ages, as well as those with injuries. Their main tasks include treatment, disease prevention, rehabilitation, and health promotion. In addition to primary care physicians, the centres house other specialists (rheumatology, cardiology, orthopaedics, gynaecology), as well as psychologists, dietitians, medical practice assistants, medical practice coordinators, registered nurses, and, since 2011, several APNs. In tandem with primary care physicians, the APNs from the different MediZentrum provide continuous care for LTCF residents [29, 33]. Our study focuses on one MediZentrum and one LTCF in the Bernese Seeland region. The studied centre employs two APNs who care for LTCF residents. The APNS and primary care physicians are responsible for approximately 98% of the LTCF’s residents, with the remainder receiving care from nearby practices. With roughly 100 staff, this LTCF contains more than 40 apartments and approximately 65 resident rooms, accommodating over 100 residents in total. The MediZentrum and the LTCF are not linked organizationally.

### Study sample and procedures

The current study was conducted as part of an overarching participatory research examining the APNs’ work in the Medizentrum centres (cf. [29, 33]). The participatory approach included the co-design of the research questions and methods with a local APN who also facilitated contact with the LTCF.

Recruitment of participants for primary data collection in the LTCF and from among the APNs in the MediZentrum centre collaborating with the LTCF followed a purposeful sampling approach. In the LTCF, a total of 11 potential staff were approached for focus group or individual interviews; however, four persons could not participate due to scheduling conflicts. On the LTCF side, only staff who had direct contact with the APNs in their daily work or who were interacting with APNs at an oversight level were included – we strove for heterogeneity in the experience of working with the APNs (i.e., level of collaboration, years of experience) and reached the diversity envisioned. On the Medizentrum centre side, the two APNs with a minimum employment duration of three months and German language proficiency were included for the individual interviews. Both APNs approached agreed to participate. Physicians were not approached, since data from a former study existed.

Secondary data were obtained from two former studies with the Medizentrum centres. The first study focused on APNs’ activities in primary care [29]. The local responsible APN had recruited all four APNs working at that time at four Bernese Seeland medical centres (the fifth centre did not yet have an APN) for individual interviews and a focus group, the transcripts of which were used in this study. The second study focused on task shifting between APNs and physicians in the same Medizentrum centre included in the current study (one of the four in the Bernese Seeland) [33]. Six physicians, who had at least 9 months of experience in collaboration with at least one APN working in the same Medizentrum centre were included for interviews, one of them a geriatrician working closely with APNs in the care for LTCF residents. The transcript of the interview with the geriatrician was included in the current study as secondary data since it was rich in information about collaboration with APNs in the care for LTCF residents. All participating healthcare professionals from the centres had worked for a minimum of nine months in one of the centres.

### Data collection

Primary data collection took place from October 2021 to November 2021. The second author performed this task while working as an APN in a private hospital. She conducted semi-structured individual interviews with four LTCF staff members with oversight tasks (i.e. director of nursing and ward supervisors) and the two APNs, as well as one semi-structured focus group interview with three LTCF staff members (i.e., registered nurses and licensed practical nurses; see interview guides in Supplementary File 1). The interview guide for the individual interviews was tested in advance with a fellow student from the University of Basel who worked as an APN, after which no further adjustments were made. Before data collection, the second author performed a work-shadowing of one APN’s work. The resulting observations were used to familiarize the researcher with the setting but were not integrated into this study. The second author had no personal contact with the other study participants before or after the study. Participants were informed before the interview that she was conducting the study as part of her master’s thesis.

Interviews took place in a meeting room at the LTCF. Individual interviews ranged from 22 to 53 min in duration, and the focus group interview lasted 58 min. Only the participants and the second author were present during the individual interviews. The focus group interview was co-moderated by a graduate student in nursing. All interviews were recorded as audio files and transcribed in standard German. The study group’s demographic data were collected using a questionnaire.

Secondary data collection took place between August 2019 and February 2020 as part of two previously published master’s theses [29, 33]. A single interview was conducted with a geriatrician [33] and four individual interviews with APNs, followed-up by phone calls during the analysis phase [29], along with a single focus group interview involving all four APNs [29]. Interviews lasted between 87 and 89 min; phone calls lasted between 26 and 33 min. As with the primary data, interviews and phone calls were recorded as audio files and transcribed in standard German, and demographic data were collected using questionnaires.

### Data analysis

For data analysis, both primary data and secondary data (i.e., transcripts from the former studies) were taken together as basis to follow Braun and Clarke’s iterative six-phase process of an inductive thematic analysis [31]. Data from the former studies were re-analysed and no codes were re-used. After familiarisation with the data (phase 1), the primary data were coded followed by the secondary data (phase 2) and codes with corresponding texts were collated to identify important patterns (phase 3). Potential themes were built and reviewed within the research team, re-reading the data and further developing the codes (phase 4, see Supplementary File 2). Themes were then checked against the research questions and checked for coherence and consistency (phase 5), before writing up and extracting citations to support the themes (phase 6). MAXQDA software was used to support data coding [34]. The data were coded by the second author. While the first research question about APNs’ tasks was approached in a descriptive way, the second about the collaboration was explored with a reflexive thematic approach. Quality assurance measures for the entire research process included regular discussions and reflections within a peer group of master’s students in the second author’s master’s seminar in the Institute of Nursing Science at the University of Basel. Additionally, two experienced qualitative researchers with backgrounds in nursing and ethnology provided guidance on the analytical processes, discussing each step of the process and giving feedback and reflecting together on codes and themes. Some data redundancy between statements of APNs in the primary and secondary dataset was observed, pointing towards data saturation.

### Ethical considerations

Due to the small sizes of the MediZentrum’s interdisciplinary teams, complete anonymization of the data was not possible, i.e. the identity of the APNs involved was open for all persons involved in the data analysis. All confidential information was pseudonymized. Regarding ethical considerations, the Cantonal Ethics Commission of Bern determined that this study does not fall under the Human Research Act (BASEC-No. Req-2021–00537).

## Results

### Participants

The sociodemographic data of the study group from the primary data collection (*n* = 7 LTC staff and *n* = 2 APNs) are shown in Table 1. LTC staff had at least every two to three weeks contact with an APN.

**Table 1:** Sociodemographic data of the primary data collection study group

	**Primary dataset**		**Secondary dataset**
	LTCF Staff (*n* = 7)	APNs (*n* = 2)	APNs (*n* = 4)
**Gender**			
female	6	2	4
male	1	-	-
**Age**			
31 – 40	-	1	3
41 – 50	3	1	1
51 – 65	4	-	-
**Highest Educational Qualification**			
Upper secondary vocational training^a^	1	-	
Short-cycle tertiary vocational education^a^	6	-	
University of Applied Sciences (Master)	-	1	3
University (Master)	-	1	1
**Employment Percentage**			
10 – 40%	1	-	-
40 – 70%	1	2	2
80 – 100%	5	-	2
**Work Experience (in years)** **(from the first educational qualification in healthcare)**			
6 – 10	-	1	Not available
11 – 15	-	-	
16 – 20	1	-	
21 – 25	-	1	
26 – 30	5	-	
Over 30	1	-	
**Frequency of Direct Contact with an APN**			
Daily	1	-	-
3 – 4 times per week	2	-	-
1 – 2 times per week	2	-	-
every 2 to 3 weeks	2	-	-

^a^According to International Standard Classification of Education (ISCED). The upper secondary vocational training is a 3-year education similar to licensed practical nurses, the short-cycle tertiary vocational education is a 3-year vocational training as registered nurse

Participants in the secondary data collection were all female and between 26 and 64 years old. All had been employed at one of the centres from one to eight years, with percentages of full-time employment ranging from 60 to 90%. The APNs had 3 to 23 years of nursing experience and held master’s degrees in nursing (representing a minimum of 90 European Credit Transfer System (ECTS) points, where 1 ECTS corresponds to 30 h of study).

### Tasks of APNs in LTCFs and building blocks of collaboration

Based on the results of our primary and secondary data, our results reflect the experiences of the participating APNs and their colleagues both in their medical practice and their LTCFs. The first theme, “Taking on tasks in Resident Care,” addresses the first research question about APNs’ care tasks, providing an insight into the APNs’ daily practice. How the collaboration between APNs and LTCF staff regarding resident care is structured is explained in the second theme, “Laying the Foundation for Collaboration.” Three subthemes elaborate key aspects of collaboration —“Working Together to Clarify Roles and Responsibilities,” “Determining Types and Paths of Communication,” and “Building and Maintaining a Trusting Relationship” —which then allow for the third theme, “Working Together to Achieve the Best for Residents.”

### Taking on tasks in resident care

APNs undertake a wide range of tasks related to resident care. They serve as the main point of contact for both nursing and medical questions from residents, their families, and LTCF staff. APNs are familiar with all residents who receive medical care from the MediZentrum and take on case management in stable situations, while the geriatrician focuses on unstable situations.

All participants agreed that the APNs’ tasks focus on their weekly APN visits. Each visit involves a meeting between the APN and the daily supervisor on the wards in the LTCF, during which residents’ situations are discussed (based on written documentation) and any open questions addressed. Based on the documentation provided, APNs recommend adjustments to therapies (e.g., increasing or decreasing analgesia, continuing anticoagulation) and request laboratory tests within their competencies. After this discussion, APNs visit residents and conduct clinical assessments for further information gathering. Based on the information gathered from each resident meeting, the APN suggests any necessary adjustments to the therapy or laboratory tests. When necessary decisions fall outside the APNs’ competencies, they consult with the MediZentrum’s primary care physicians, who bear primary responsibility for residents’ medical care.

Participants reported that APNs predominantly perform tasks relating directly to clinical practice. As examples, they perform ear irrigations, administer infusion therapies, and assist LTCF staff in difficult blood draws. According to APNs, their roles also involve both teaching and coaching. They instruct and supervise staff in complex nursing situations, such as exacerbated pain situations. APNs also supervise palliative situations, assess wound or skin conditions as needed and show staff how to administer appropriate treatment, such as wound dressing. Staff members reported that they contact APNs when they reach their limits in treatment and require professional support. APNs reported that they instruct staff based on current evidence and verify the latest evidence-based practice, as well as considering how to connect and integrate such findings into the LTCF’s daily practice.

Another APN duty is to maintain contact with residents’ families. They do this not only when developments require it, e.g., when residents show changes in their condition, but also at the request of the residents and/or their families. This is part of APNs’ aim to support residents and their families to make informed decisions regarding possible therapies or hospitalizations. Their focus is on maintaining residents’ quality of life. They also regularly discuss residents’ current health situations with primary care physicians, then coordinate the necessary therapies across service providers. To execute APNs’ recommended tasks, i.e., to provide advanced services to residents, participating care staff reported that effective collaboration among team members is essential.

### Laying the foundation for collaboration

#### Working together to clarify roles and responsibilities

All interviewees recalled that, at the time the new care model was introduced, it had met with considerable scepticism. In particular, LTCF staff were unsure which tasks APNs would undertake. They suspected that it would lead to more interprofessional interfaces with the APNs as link between the LTCF and the primary care physician, thereby increasing their workload. All confirmed that this suspicion was unfounded, and that the planned collaboration had worked very well. To ensure its success, APNs emphasized that tasks and competencies—who does or can do what—must be defined from the outset. APNs noted that if they had not engaged the staff and defined all relevant competencies and tasks early, conflicts would likely have arisen. They also acknowledged that it was essential that they did not take competencies or tasks away from staff.

*[I told them,] “I won’t take anything away from you, especially those things you like to do.” And once you’ve got them on board, then it’s all good; then they’re friends.... (APN)*

In trying for a collegial atmosphere, APNs emphasize that they do not wish to appear arrogant in their roles but rather to be a part of the LTCF care team. This egalitarian relationship was considered important by all interviewees. The joint establishment of the care model—including of the individual roles and tasks—was considered important not only by the APNs but also by all other interviewed staff. All agreed both that the division of tasks and responsibilities was meaningful and that they did not wish for any changes.

#### Determining types and paths of communication

The interviewees described various communication channels between APNs and LTCF staff. The main deciding factor as to which channel LTCF staff used was the urgency of the situation. They collected every-day, non-urgent questions for the weekly APN visit. Time-sensitive questions that cannot wait for the visit are submitted to the APNs via email or telephone. The APNs’ responses or therapy adjustments are also sometimes executed via email. Initially, however, as APNs needed to clarify instructions for therapy changes with the responsible primary care physicians, they often sent these instructions to their LTCF colleagues late at night. Since staffing levels are minimal during the evening and night shifts, no one would then be available to implement the recommended changes.

All interviewees also acknowledged the importance of motivating communication in collaboration that is appropriate to the audience, i.e. uses language that is suitable to the audience’s level of understanding and provides enough context information to help the audience understand the message. This was especially true for the APNs’ coordination-focused communications. Such a positive experience was new for staff: According to interview statements, the previous care model’s communication (with primary care physicians) was occasionally neither motivating nor audience-appropriate. In collaboration, the APNs emphasized both that motivation-based information sharing is particularly important and that any changes to measures must also be justified.

APNs’ explanations of why specific tasks had to be performed were also considered essential by staff. The APNs agreed that when staff members understand the reason for a change, they are more motivated, increasing the likelihood of task completion:

*The* *nursing* *staff should also be informed in a way that motivates them to implement… and actually [make any recommended changes]. And that is very important; so, 50% [of successful collaboration] is communication. (APN)*

The ability to accurately relay information from service providers and explain the rationale behind that information to other service providers (e.g., LTCF staff), was also considered important by all interviewees. For example, when APNs pass on mobility-related information from physiotherapists to the relevant nursing staff, if that information does not flow as desired, any misunderstandings can impact the interprofessional collaboration and the quality of resident care.

As might be expected, APNs often interact with staff with lower educational levels. Accordingly, not all staff members are familiar with medical terminology, and few have tertiary-level education. To prevent misunderstandings, then, it is often necessary for APNs to adapt the medical vocabulary to more accessible terms. To facilitate information flow and effective collaboration, APNs expressed a desire to have more staff with tertiary education in the LTCF as counterparts.

#### Building and maintaining a trusting relationship

All participants reported that a healthy relationship is based on trust. However, mutual trust must be built and does not exist from the beginning.

*All right, but we can rely on them, and we get an answer, a smart one, to our* *question* *. Through this, trust has developed, and that’s actually the basis... so that has naturally developed over the course of our collaboration.” (LTCF Staff Member)*

All interviewees agreed that APNs had earned their trust over the course of their collaboration, and that trust can be considered the basis of collaboration. Collaboration was perceived as good, humane, and cooperative by all staff members. APNs reported that they had to earn this perception from each individual staff member.

*Nowhere is there greater competition than in nursing itself. And you have to reach* *out* *to each individual and prove to them, come on, we’ll do it together. (APN)*

APNs explained that trust was built gradually, and they had to prove to staff that they shared the same goals. By the time of data collection, LTCF staff narrated that most LTCF staff members considered the APNs members of their team, not as external entities. This trust must be mutual: APNs must also be able to trust staff. According to the APNs, instructions, such as measuring vital signs like blood pressure and body weight, are sometimes not carried out accordingly. However, if a staff member fails to take the measurements the APN has specified, APNs described increasing workload, since they needed to check every time whether the necessary data have been collected. Therefore, the APNs commented that they would like to see an improvement in the implementation and compliance with standard measurement practices by staff.

### Working together to achieve the best for residents

Under the new APN-physician tandem care model, all participants rated the care of residents as qualitatively better than before its introduction. The majority of the interviewed staff considered the APNs an asset for the residents as well as for themselves.

*So* *, in that sense, I think the quality for the residents is good. It’s really very good. (Staff member, LTCF)*

In particular, the staff positively evaluated the improved level of consistency regarding resident care. The APNs visit weekly and know each resident. They take time for the residents as well as for the staff. According to the staff, the primary care physicians did not provide the same consistency in their care as the APNs. They were also less familiar with the residents and spent less time with them. The staff members noted that they are more likely to bring up questions in collaboration with the APNs than with the primary care physicians under the previous model. To them, the threshold for collaboration with these nurses is considerably lower:

*Yes, I have the feeling that we dare to ask questions earlier, where we used to wait a bit longer. And we found that has to be the doctor now... and now we say rather, oh, it’s not bad if someone else looks at it.... (Staff member LTCF).*

Bringing up questions earlier leads to earlier opportunities to address or even prevent problems in residents. Collaboration is viewed by all as working together rather than simply side by side, as was previously often the case with primary care physicians. With the APNs, ongoing exchanges and maintenance of their relationships with care staff and residents alike can be achieved through their weekly visits. Overall, for the APNs, this care model enables proactive rather than problem-oriented action.

## Discussion

This qualitative research explored APNs role based at a multiprofessional primary care centre and their collaboration with LTCF. When caring for LTCF residents, APNs perform the same / similar tasks as primary care physicians. They lead case management during weekly visits and in the interprofessional team. The LTCF staff perceive the collaboration with these APNs as very good. However, to establish a durable, constructive collaboration, it is vital that roles and tasks in the interprofessional team are carefully defined. Mutual trust must be built and continuously maintained. Together, then, the APNs and their LTCF staff can achieve the best possible outcomes for the residents.

### Tasks of APNs in LTCFs

The APNs’ tasks in this study are focused on direct clinical activities such as the weekly APN visit, the subsequent clinical assessments of residents, and the resulting measures. Administratively, APNs’ regular presence strengthens the coordination of health services and continuity of care [35]. In addition to direct clinical practice, APNs concentrated on supervising and coaching LTCF staff, similar to international practice [35–37]. In general, considering the examined APNs’ setting, main tasks and competencies, their roles correspond closely to those of NPs with the addition of tasks in quality development [14].

Switzerland is in an early phase of introducing APN roles in primary care. Given the lack of regulation for autonomous practice, APNS are typically part of the physician team and do not work in an independent practice [25]. As we have seen in our study, being embedded in the multiprofessional team allows APNs to closely collaborate with physicians, with whom they share their documentation and have regular case discussions [33]. Internationally, Lovink, Laurant and colleagues [8] report similar benefits from APNs collaboration with primary care physicians as part of a multiprofessional team, noting that the APNs were able to substitute most of the physicians’ activities. However, there are other models, as in Australia, where legislation requires that APNs collaborate with primary care physicians, without having to be part of an interprofessional team [38].

One internationally-noted benefit of APN activity is staff training and quality development [8, 9, 35, 38, 39]. APNs’ focus on coaching and training of staff nurses can catalyse development of those nurses’ clinical skills, boost their confidence, and increase the associated quality levels [13]. This kind of support also bolsters staff job satisfaction [35]. However, it also creates a dilemma: while staff development is vital to ensure the quality of care, APNs must limit the amount of support they provide, as their time/funding is limited.

According to Ervin, Reid and colleagues [38], the lack of secure ongoing funding for care models that rely on APNs threatens both the implementation and the sustainable implementation of APN care in LTCFs. For example, as APNs in most parts of Switzerland are not authorized to bill for their services, physicians must both recognize their added value in LTCF resident care and be willing to engage in unconventional financing models. Despite the high demand for APN services in primary care, then, systemic complications involving autonomy and remuneration can make the work less appealing.

### Collaboration with LTCF staff

As discussed in the literature, for care models with APNs to be successfully implemented and the associated interprofessional collaboration launched, the APNs’ roles, tasks, competencies and expected outcomes must be clearly defined and known to all involved parties [37, 38]. Moreover, these points must be understood *before implementation*.

As a recent Australian study showed, careful communication of the rationale for introducing APN roles is important to their overall acceptance. In that study, uninformed LTCF staff nurses initially assumed that a nurse practitioner’s (NP’s) arrival was a sign that they were not doing their jobs adequately [38]. This misunderstanding led to a reduction of staff skills, as care providers became unsure about what they were allowed to do. Similarly, the APNs in our study reported that their LTCF’s staff took time to accept that they were not there to take anything away from the staff, but to facilitate residents’ access to medical services. Accordingly, the APNs took on the job of clarifying their roles and tasks. Lovink, Laurant and colleagues [8] also emphasized the importance of proactive approaches and clear communication to care teams. The LTCF staff are important stakeholders in the successful introduction of the APN role.

In the literature, effective communication and mutually trustful relationships with the other interprofessional team members are considered key factors for collaboration [35, 37, 39]. Building trust takes time [38]; however, structures such as regular visits can support this process. As we could show, in addition to demonstrating that they are reliable and competent, provide accurate information and take employees seriously, APNs must avoid asserting hierarchical privilege— “pulling rank”—in their interactions. This need to prove themselves is also described in an Australian study [38]. One advantage APNs in our study had, was their ability to speak the languages both of doctors and of nurses. This allows them to act as liaisons between the two professional groups, while being particularly accessible to nursing staff. The same dynamic has also been observed in other countries [35].

Overall, by being embedded in a multiprofessional team at the medical centre, APNs serve as a bridge between LTCF employees and physicians [38]. While APNs facilitate access to GPs, their dependence on physicians’ decisions can affect the timeliness of communication. For example, LTCF employees in our study wished for faster feedback after visits; however, this is not always possible when APNs must first consult with the responsible physician due to their competency limits. A similar feedback issue was noted in a Dutch study, which found that APNs take longer than doctors for clinical reasoning [8].

We saw that APNs also need to trust that their care teams will implement their instructions and recommendations. When this does not happen, it leads to delays, increased workload for the APNs (e.g., asking back, double-checking, return another time, if resident was not ready for an exam) and ultimately a decline in the quality of care. A similar challenge was reported by NPs in a US study, who expressed frustration that their prescriptions were not reliably implemented [39]. However, this seems to be less related to the fact that it is an NP who is prescribing, but to internal structures in the LTCF, as similar problems are also described by GPs.

### Improved outcomes

Once the foundation is laid for effective collaboration, we saw that staff and APNs can work together to achieve the best possible outcomes for the residents. A recent US study’s results showed that employees perceived a quality improvement through care models with APNs [39]. Numerous others have found that, as LTCF staff members collaborate with APNs, their residents’ care-related outcomes improve [4, 8, 13, 17–19, 21–24, 26, 27].

In our study, we did not hear about specific outcomes that were improved. Rather, it was the improved processes of collaboration that allowed for more person-centred care, reaching outcomes important to the resident. Together, APNs and LTCF staff in our study covered nursing, medical, and social issues, i.e., all three major dimensions of resident care. Through their regular visits, APNs became familiar with residents and staff, enabling them to assess what is possible and what is not in case of deteriorating health. The APNs were more accessible for LTCF staff than the primary care physician, not only because they used language that the staff members could understand, but are also because they were more readily available. It has been shown before that for LTCFs, it is often difficult to reach GPs in emergencies, which can lead to avoidable hospital admissions [38, 40]. With the timely availability of APNs who are familiar with the residents’ situations, initial responses, triage, and on-site treatment can all be initiated more quickly [36, 38]. For long-term care residents, this allows person-centred, individualized care, and the maintenance of the best possible quality of life [8, 13, 41], which was also at the core of what our study found.

### Strengths and limitations

A strength of this study is the multiple perspectives included, allowing to increase the internal validity of the results. Due to the participatory approach, two employees of the medical centres are also co-authors of this study. Neither was involved in data analysis, but the findings were checked with them and they added a high contextual understanding to further increase that validity of the results. In addition, the researcher was an APN familiar the challenges of role development and collaboration with nursing teams. On the other hand, a limiting factor is that there was no consultation about the findings with LTCF staff. A further limitation is the very specific setting: The study describes one practice, which limits the sample size, focusing on the collaboration of one MediZentrum with an LTCF in a healthcare system where APNs’ roles in primary care are still being established. This is a model still developing. However, the studied primary medical care practice has the longest experience working with APNs and has been able to consolidate that model over several years. While transferability to other healthcare systems outside of the studied medical centres cannot be guaranteed, similar themes regarding APNs tasks and their collaboration with LTCFs can be found in the literature. Accordingly, results might have an external validity, allowing for a careful transfer to primary care with similar setups. Last, the study focuses on the collaboration of APNs and LTC staff and touches little on the perspective of physicians; this is focused in a former article that addresses the changes in the professional roles of GPs with the introduction of APNs in a multiprofessional primary care practice [33].

### Implications for practice, policy, and research

To introduce APN-physician tandem models in practice and ensure productive collaboration between APNs and LTCF staff, it is recommended to jointly establish the care model and carefully implement the APNs’ roles, using for example the PEPPA plus Framework [42]. This will help to clarify roles and expectations before implementation. At policy level, both the legislation about APN competencies and securing financing are key to promote such care models and profit from their effect on resident outcomes.

The integration of APNs into multiprofessional teams in Switzerland’s primary medical care and related care models, i.e., those that provide LTCFs access to APNs, should be further explored. For any such exploration, to clarify reinforcing or hindering factors, it is important to describe not only the APN model itself but also its underlying medical care model [33, 43]. In addition, to facilitate inter-study comparison and increase transferability, meaningful standardized quality indicators should be developed to evaluate new care models.

## Conclusion

The insights gained from this study provide an overview of the everyday practices of Swiss APNs in the care of residents in LTCFs. Our observations indicate that the studied APNs’ tasks are diverse, and that they provide multidimensional person-centred care to residents in collaboration with LTCF staff. Together, they address nursing, medical, and social issues, i.e., all three dimensions of resident care. Our findings indicate that, under the studied APN-physician tandem care model, the LTCF’s residents receive qualitatively better care than with physicians only, due to the more pro-active approach to resident problems and the better access to medical care when the residents’ health status changes, which also increases the staff’s satisfaction levels.

### Supplementary Information

Supplementary Material 1.

Supplementary Material 2.

## Data Availability

The datasets generated and/or analysed during the current study are not publicly available due to confidentiality issues (given that the setting of the study is known and the limited number of participants, data is traceable) but are available from the corresponding author upon reasonable request.

## Abbreviations

APN: Advanced Practice Nurse
CNS: Clinical Nurse Specialist
GP: General Practitioner
LTCF: Long-term care facility
NP: Nurse Practitioner
UK: United Kingdom
USA: United States of America
WHO: World Health Organization
