# Supplementary material for: Advanced practice nurses’ daily practices delivering primary care to residents in long-term care facilities: a qualitative study
Source: BMC Prim Care. 2024 Jun 8;25:203. doi: 10.1186/s12875-024-02455-9 (PMC11161973; doi:10.1186/s12875-024-02455-9)
Supplement: Supplementary file 1 — Supplementary Material 1. [file 12875_2024_2455_MOESM1_ESM.docx]

**Advanced practice nurses’ daily practices delivering primary care to residents in long-term care facilities: A qualitative study – Supplementary File 1**

**Interview guide for individual interviews and focus group with healthcare professionals**

| **General deepening and maintenance questions** | | |
| --- | --- | --- |
| - Why don't you tell me a little more about it - Can you describe .... in a little more detail? - What happened next? - How was that exactly? - What happened after that? - I'm not sure if I've understood this correctly, can you explain it to me again? | | |
| **Opening question** | | |
| Can you tell me about your last encounter with the APN? | | |
| **Key questions** | **In-depth questions** | **Possible topics** |
| Where do you have further encounters with the APN? | - For which activities? - Where do you have direct contact? - Where do you have indirect (telephone) contact? - Where are the main intersections? - What activities does the APN carry out on a daily basis? - How do the residents react to the APN? | - Visit - Family discussions - Exchange - Training - Consulting |
| In which situations do you contact the APN in your everyday life? | - What changes do you contact us about? - What questions do you get in touch with? | - Emergency situation - Medical changes |
| Where do you have encounters with the family doctor at the MediZentrum? | - For which activities? - Where are the main intersections? - How often does this happen? - When do you have indirect (telephone) contact? - When do you have direct contact? | - Visit - Family discussions - Exchange - Medical questions |
| What is your experience of working with the APN? | - What is your experience of communication? - What do you observe regarding time management? - What challenges do you face? | - Accessibility - Acceptance - Responsibilities - Time - Communication |
| If you had one wish, what would you wish for in terms of cooperation with the APN?  ... in relation to the activities of the APN? | - What suggestions for improvement come to mind? - What additional activities could the APN take on, what could someone other than the APN do? | - Ideas - Suggestions |
| **Final question** | | |
| I have now asked all my questions, is there anything else you would like to say that we have not addressed so far? | | |

**Interview guide for individual interviews with APNs**

| **General deepening and maintenance questions** | | |
| --- | --- | --- |
| - Why don't you tell me a little more about it - Can you describe .... in a little more detail? - What happened next? - How was that exactly? - What happened after that? - I'm not sure if I've understood this correctly, can you explain it to me again? | | |
| **Opening question** | | |
| How do you experience a typical working day as an APN in retirement and nursing homes? | | |
| **Key questions** | **In-depth questions** | **Possible topics** |
| Are there other activities that do not occur in a typical working day? | - What else do you do? - What diagnostic tools do you use? - What nursing activities do you carry out? - Which residents do you look after? | - Investigations - Laboratory diagnostics - Conversations - Visit - Training - Coaching |
| You have told me about encounters with xy.  Is that typical of your work in long-term care facilities? | - Who else do you have contact with? - How does the contact take place? - Where does the contact take place? - What is discussed? | - Telephone rounds - Medical questions - Exchange |
| In what situation do you contact the MediZentrum family doctor? | - For which problems? - What activities do you need to discuss with the doctor? - What topics do you need to discuss with your doctor? | - Emergency situation - Medical changes - Competencies |
| What is your experience of working with the various specialists at the long-term care facility? | - Where do the interfaces take place? - What are the challenges? - What are the positive points? | - Interdisciplinary cooperation - Responsibilities |
| You mentioned before that you have contact with the residents. How do you react to them? | - What activities do you carry out that are not performed by others? - How long can you spend with the residents? | - Time - Contact person - Trust - Continuity |
| What differences do you see in care when residents are looked after by the family doctor instead of you? | - How much time does the doctor have with the residents? - What activities does the doctor perform that you do not? | - Time - Trust |
| If you had two wishes, what would be the first thing you would wish for in terms of cooperation with professionals in long-term care facilities?  Secondly, what would you wish for in terms of your role and its development? | - What suggestions for improvement come to mind? - How should your role develop? - What should happen socially and politically? | - Role development - Political regulation - Cooperation |
| **Final question** | | |
| I have now asked all my questions, is there anything else you would like to say that we have not addressed so far? | | |

**Interview guides from Lauber et al.**

Lauber, E., Kindlimann, A., Nicca, D., Altermatt-von Arb, R., Sgier, C., Staudacher, S., Sailer Schramm, M., Vökt, F., & Zúñiga, F. (2022). Integration of an advanced practice nurse into a primary care practice: A qualitative analysis of experiences with changes in general practitioner professional roles in a Swiss multiprofessional primary care practice *Swiss Medical Weekly*, *152.* https://doi.org/10.4414/smw.2022.w30199

**Interview guide for individual interview with physician**

| Date: |
| --- |
| **Key question / narrative prompt** |
| ***Can you please tell me what your day-to-day work with the APN looks like?*** |

| **Key questions** | **General deepening and maintenance questions** | **Possible topics** |
| --- | --- | --- |
| General deepening and maintenance questions:   - Tell us a little more about it - Can you ... describe it in a little more detail? - What happened next? - What was it like for you? - What else can you think of? - I am not sure if I have understood this correctly, could you please explain again? | | |
| **How has your day-to-day work changed since the introduction of the APN?** | - What is easy? - What is difficult? - What's new? - How have processes changed? - Can you describe specific situations? - What is your experience of working with the APN? | - Team dynamics - New resources - Traditional hierarchy - Responsibilities |
| **How have your tasks changed in recent years?** | - What new activities have you added that you didn't do before? - Which activities have you handed over? - Has anything else changed for you apart from handing over activities? - Are there activities that your colleagues hand over to the APN but you prefer to do yourself? Which ones? Why do you prefer to do them yourself? - Can you describe how these changes have affected your relationship with your patients? |  |
| **How have the roles in the team changed overall?** | - In which areas was it easier / more difficult before? - What is easier / more difficult now? - How does the team interact with each other? - What makes the Schüpfen treatment team/care model successful? | - Team dynamics - Responsibilities |
| **How was the collaboration with the APN for you at the beginning - how is it now?** | - What feelings did it trigger in you at the beginning? - What was it like for you at the beginning with regard to trusting the work of the APN/control for tasks that you handed over? - How is it now? | - Confidence building - Uncertainty - Influence for own role - Control |
| **How do you experience working with the APN in relation to your role as a doctor?** | - What is that feeling? - How do patients react to this? | - Authority - Acceptance - Responsibilities - Control |
| **In your opinion, does it make sense to continue employing APNs as in the MediZentrum?** | - What does it take? | - Acceptance |
| ***I have asked my questions - is there anything else you would like to say that we have not addressed so far?*** | | |
| ***Would you like to add anything else?*** | | |
| ***Then I'll close the interview now and turn off the recorder*** | | |

**Interview guides from Altermatt-von Arb et al.**

Altermatt-von Arb, R., Stoll, H., Kindlimann, A., Nicca, D., Lauber, E., Staudacher, S., Sailer Schramm, M., Vökt, F., & Zúñiga, F. (2023). Daily practices of advanced practice nurses within a multi-professional primary care practice in Switzerland: a qualitative analysis. *BMC Primary Care*, *24*(1), 26. https://doi.org/10.1186/s12875-023-01977-y

**First interview with APN**

| **General deepening and maintenance questions** |
| --- |
| - What other aspects are there, for example based on a situation you have experienced? - Why don't you tell me a bit more about it? - Can you describe .... in a little more detail? - And then? - What was that for you? - How was that exactly? - What happened after that? |

| **Key questions** | **In-depth questions** | **Background to the question** |
| --- | --- | --- |
| What does a typical working day look like for you? | What does another typical working day look like for you?  If you look at your day from morning to night, what does it look like?  I'm interested in all your activities, tell me a bit more about them.  What additional activities does your working day involve?  What mundane tasks are part of your everyday work?  What would you like to have more time for during your work? What do you miss out on?  What helps you to implement your activities in the way you envision? Can you give me a concrete example?  What prevents you from carrying out your activities in the way you envision? Can you give me a concrete example?  What could be improved?  What patient and family needs do you recognize that are not yet being met? | Research question, social constructivism, background proposal |
| How do you experience your work? | When you think back to the time when you started at the MediZentrum, how did you experience the work then and how do you experience it now?  How have your activities changed since you started working here at the MediZentrum?  You work with different professional groups. How do you experience the division of who does what?  Which activities would you like to take on? Hand them over? Which activities would you like more support with?  Are you experiencing mentoring? What does this mentoring look like? Structural support? Do you participate at a strategic level of the MediZentrum? How does the number of patients influence your experience?  Personal characteristics, knowledge, skills, values of the APN  What is it that makes your day-to-day work so enriching?  What is it that makes you experience your day-to-day work as stressful?  Your work is not only shaped by your immediate surroundings here at the MediZentrum, but also by external environmental factors. How do you experience their influence, e.g. the financing mechanisms? What other environmental factors influence your work? What about health policy, outcome evaluation and performance improvement, marketing and contracts, organizational structure and culture, regulatory requirements and demands, and business aspects? | Research question,  Social constructivism: historical experiences, background Proposal |
| How do you experience your work in the environment of the Bernese Seeland MediZentren? | How do you deal with each other as the Schüpfen/Täuffelen/Messen/Lyss team?  How does this influence your activities?  What else is part of the team culture?  Who do you interact with on a day-to-day basis?   - Patients - Relatives - Doctors - How do you experience working in tandem? - Carers (APH, Spitex etc.) - Medical practice assistants   What is your experience of working with them?  How would you describe your role?  What makes the Schüpfen/Täuffelen/Messen/Lyss care model successful?  What does the supply model need to continue to be successful in the future?  What would be different if you were an APN in the UK/USA/Canada/Australia?  What are your answers based on? Have you had your own experiences? | Research question,  Social Constructivism: Culture, Interactions, Background Proposal |
| What impact do you **expect** your work and presence to have on patients and their relatives? | What do you want to achieve with your work?  What would you like to achieve with ...*insert activity..*.?  How do you achieve the desired effect?  How do you think you can achieve this?  With the patients?  With the relatives? |  |
| What effects of your work and presence do **you recognize/notice** in the patients and their relatives? | How do you recognize that it was successful/unsuccessful?  With the patients?  With the relatives?  What factors influence the results achieved?  What experience have you had with activity/strategy XY?  What was successful?  What was not successful?  What are your greatest strengths in making a difference to your patients and their families?  What feedback have you received from patients about activity XY?  How do you get feedback on your work as an APN? | Research question, background proposal |
| How do you **experience** the effect of your work and presence on patients and relatives? | How do you experience the effects of your work and presence in challenging patient and family situations?  What effects on patients/relatives motivate you to continue this activity? | Research question |
| **Final question** | | |
| We have discussed various aspects of your activities in the context of the MediZentrum Schüpfen/Täuffelen/Messen/Lyss, how you experience the activities and what effects you expect, perceive and experience on patients and relatives. Is there anything else you would like to add from your point of view, anything I haven't thought of? | | |

**Follow-up interview with APN**

| **General deepening and maintenance questions** | |
| --- | --- |
| - What other aspects are there, for example based on a situation you have experienced? - Why don't you tell me a bit more about it? - Can you describe .... in a little more detail? - And then? - What was that for you? - How was that exactly? - What happened after that? | |
| **Key questions** | **In-depth questions** |
| Refer to observed activities, statements, effects, context | - I have seen *this activity*. With what background did you do/say this? - When you do *activity XY,* what do you think it does? - How do you think this is perceived by colleagues/patients/relatives/external professionals? - What is visible to the outside world and what is not? |
| What aspects of the first interview are still open? | - Activities - Experiencing the activities - Expected effects - Effects experienced - Context - What would you like to deepen? |
| Aspects from the previous interviews and Go-Alongs that I would like to expand on | - Activities/ Effects/ Context/ What was said/ Observed/ Heard/ Question |
| **Final question** | |
| We have various aspects of your activities in the context of the MediZentrum Schüpfen/Täuffelen/Messen/Lyss  how you experience them and what impact they have on patients and relatives. Also, what effect you have on patients and relatives. Is there anything else you would like to add from your point of view, anything I haven't thought of? | |

**Focus group with APNs**

| **Introduce topic** |
| --- |
| The aim of this study is to understand what you do as APNs, what your activities involve and how your presence and work affect patients and relatives from your perspective. All this in the context of the MediZentren of the Bernese Seeland. |
| **Introductory question** |
| When you see these topics, what goes through your mind? |
| **Further questions** |
| How do you see the research question "How do APNs experience their activities and their impact on patients and relatives in the context of the Medizentren of the Bernese Seeland?" answered with these topics?  Do these topics reflect your reality?  Which theme fits best? Why?  Which topic raises questions?  Which topic fits the least? Why?  How would you adapt/refine the definition of *topic XY?*  What activities would you have expected that are not listed?  What effects on patients and relatives would you have expected that are not listed now?  What contextual factors would you (not) have expected? |
| **General deepening and maintenance questions** |
| - Can you describe this in more detail? - Can you / can others say more about this? - (after statements of positive/negative examples) Are there also contrary experiences/opinions? - What did that trigger in you? How did you react? - How was that exactly? - What do you mean?   🡪 Involve co-moderator on an ongoing basis as required |
| **Final question** |
| We have addressed various aspects of the topics. Is there anything else you would like to add from your point of view, anything we haven't thought of? |
