# Supplementary material for: Advanced practice nurses’ daily practices delivering primary care to residents in long-term care facilities: a qualitative study
Source: BMC Prim Care. 2024 Jun 8;25:203. doi: 10.1186/s12875-024-02455-9 (PMC11161973; doi:10.1186/s12875-024-02455-9)
Supplement: Supplementary file 2 — Supplementary Material 2. [file 12875_2024_2455_MOESM2_ESM.docx]

# Supplementary File 1

Advanced Practice Nurses' Daily Practices Delivering Primary Care to Residents in Long-Term Care Facilities: A qualitative study

Code tree – translated English version

| Codes | Main Themes (and Sub-themes) |
| --- | --- |
| APN as main contact person  Weekly APN Visit  Making adjustments to therapy  Clinical assessment by APN  Review of therapies and measures  Consultation between primary care physicians and the APN.  Teaching and Coaching  Evidence-based practice  Palliative care and symptom management  APN support in ethical decision making  Professional exchanges between APN and staff  APN advice regarding medical and nursing problems  Support with complex wounds and skin conditions | Taking over tasks regarding residents’ care |
| Employee scepticism at the beginning of the care system  APN as interface between all stakeholders  Scepticism about the APN role was accepted as unjustified  Role clarification  Boundaries of competencies  Shared design of APN visits  APN know how staff work and vice versa  No hierarchy between APNs and staff (or APNs and staff treat one another as equals)  Communication as the basis of all actions  Communication channel vary depending on urgency  Email exchange  Telephonic exchange  Spontaneous exchange  Various times for therapy adjustments  Therapy adjustments sometimes only communicated in the evening  Importance of motivational information exchanges  Providing rationales for measures  APN communicates more accessible than physicians  Tertiary-educated staff are rare in the home Interdisciplinary exchange  Increased workload due to poorly trained staff  Trust as the basis for collaboration  Building trust  Mutual trust  Orders are partially not carried out | Laying the foundation for collaboration  *Defining roles and duties together*  *Determining communication modes and paths*  *Establishing and maintaining a trusting relationship* |
| APN and employees pull together  Quality level higher with APN than before  Increases in consistency through the APN’s support  Inhibition threshold of employees lower with APN (than with physicians)  Being proactive and preventive  Residents better cared for under the APN care model  Holistic, multidimensional care | Working together to achieve the best for the residents |

Code tree – Original German version

| Codes | Hauptthemen (Subthemen) |
| --- | --- |
| APN als Hauptansprechperson  Wöchentliche APN-Visite  Anpassungen der Therapie tätigen  Klinische Beurteilung durch APN  Überprüfung von Therapien und Massnahmen  Rücksprache mit Hausärzten durch die APN  Teaching und Coaching  Evidence based practice  Palliative Betreuung und Symptommanagement  APN unterstützt in ethischer Entscheidungsfindung  Fachlicher Austausch zwischen APN und Mitarbeitenden  APN avisiert bei medizinischen und pflegerischen Problemen  Unterstützung bei komplexen Wund- und Hautverhältnissen | Aufgaben übernehmen in der Versorgung von Bewohner_innen |
| Skeptische Haltung der Mitarbeitenden bei Beginn des Versorgungssystems  APN als Schnittstelle zwischen allen Beteiligten  Skepsis betreffend Rolle hat sich nicht bewahrheitet  Rollenklärung  Kompetenzgrenzen  Gemeinsamer Aufbau der APN-Visite  APN und Mitarbeitenden wissen, wie sie gegenseitig funktionieren  Keine Hierarchie zwischen APN und Mitarbeitenden  Kommunikation als Grundlage aller Tätigkeiten  Je nach Dringlichkeit anderer Kommunikationsweg  E-Mail-Austausch  Telefonischer Austausch  Spontaner Austausch  Unterschiedliche Zeitpunkte der Therapieanpassungen  Therapieanpassungen teilweise erst am Abend  Wichtigkeit des motivationsfördernden Informierens  Begründung von Massnahmen  APN kommuniziert adressatengerechter als Ärzte  Tertiäre sind im Heim rar  Interdisziplinärer Austausch  Mehr Arbeitsaufwand aufgrund schlecht ausgebildeten Mitarbeitenden  Vertrauen als Basis der Zusammenarbeit  Vertrauensaufbau  Gegenseitiges Vertrauen  Auträge werden teilweise nicht ausgeführt | Die Grundsteine für die Zusammenarbeit legen  *Rollen und Aufgaben gemeinsam klären*  *Kommunikationsarten und – wege bestimmen*  *Vertrauensvolle Beziehung aufbauen und pflegen* |
| APN und Mitarbeitenden ziehen gemeinsam an einem Strick  Qualitätslevel mit APN höher als vorher  Mehr Konstanz in der Betreuung durch die APN  Hemmschwelle der Mitarbeitenden bei APN niedriger  Proaktiv und präventiv tätig sein  Bewohner_innen durch das Versorgungssystem besser betreut  Ganzheitliche und mehrdimensionale Betreuung | Gemeinsam das Beste für die Bewohner_innen erreichen |
